# *VLF*: An R package for the analysis of very low frequency variants in DNA sequences

**DOI:** 10.3897/BDJ.11.e96480

**Published:** 2023-01-26

**Authors:** Jarrett D. Phillips, Taryn B.T. Athey, Paul D. McNicholas, Robert H. Hanner

**Affiliations:** 1 School of Computer Science and Department of Integrative Biology, University of Guelph, Guelph, Canada School of Computer Science and Department of Integrative Biology, University of Guelph Guelph Canada; 2 Stollery Children's Hospital, Edmonton, Canada Stollery Children's Hospital Edmonton Canada; 3 Department of Mathematics and Statistics, McMaster University, Hamilton, Canada Department of Mathematics and Statistics, McMaster University Hamilton Canada; 4 Biodiversity Institute of Ontario and Department of Integrative Biology, University of Guelph, Guelph, Canada Biodiversity Institute of Ontario and Department of Integrative Biology, University of Guelph Guelph Canada

**Keywords:** DNA barcoding, frequency matrix, genetic diversity, PCR error, sequencing error, trace file

## Abstract

Here, we introduce *VLF*, an R package to determine the distribution of very low frequency variants (VLFs) in nucleotide and amino acid sequences for the analysis of errors in DNA sequence records. The package allows users to assess VLFs in aligned and trimmed protein-coding sequences by automatically calculating the frequency of nucleotides or amino acids in each sequence position and outputting those that occur under a user-specified frequency (default of *p* = 0.001). These results can then be used to explore fundamental population genetic and phylogeographic patterns, mechanisms and processes at the microevolutionary level, such as nucleotide and amino acid sequence conservation.

Our package extends earlier work pertaining to an implementation of VLF analysis in Microsoft Excel, which was found to be both computationally slow and error prone. We compare those results to our own herein. Results between the two implementations are found to be highly consistent for a large DNA barcode dataset of bird species. Differences in results are readily explained by both manual human error and inadequate Linnean taxonomy (specifically, species synonymy). Here, *VLF *is also applied to a subset of avian barcodes to assess the extent of biological artifacts at the species level for Canada goose (*Branta canadensis*), as well as within a large dataset of DNA barcodes for fishes of forensic and regulatory importance. The novelty of *VLF *and its benefit over the previous implementation include its high level of automation, speed, scalability and ease-of-use, each desirable characteristics which will be extremely valuable as more sequence data are rapidly accumulated in popular reference databases, such as BOLD and GenBank.

## Introduction

The ability to distinguish between sequence disparity arising from true biological variation versus that arising as a result of sequencing artifacts, known to occur during the PCR/sequencing process, is of great importance. Numerous studies have noted the detrimental effect of sequencing errors on the accurate estimation of key population genetic parameters for assessment of genetic diversity, such as effective population size (*N_e_*), haplotype diversity (*h*) and nucleotide diversity (\begin{varwidth}{50in}\begin{equation*}
            \pi
        \end{equation*}\end{varwidth}) (Cummings et al. 2010, Liu et al. 2010). Both amplification and sequencing artifacts can lead to inflation of *N_e_* and standing genetic diversity, thereby challenging studies involving species of conservation importance with small census population sizes for instance (Cummings et al. 2010). In fact, this group in particular is expected to possess lower levels of nucleotide diversity as a result of the influence of genetic drift and selective sweeps acting on at-risk species populations at the genomic level (Petit-Marty et al. 2021) in comparison to non-threatened taxa. 

Concerning PCR errors, whose magnitudes are highly variable (Potapov and Ong 2017), at least one is expected to occur in upwards of 10% of amplified DNA fragments as small as 250 bp (Cummings et al. 2010). Simple extrapolation, assuming a baseline PCR error rate of 10%, might even suggest a rate of up to 26% for short, low-quality segments from genomic markers like the 5’ terminus of the cytochrome *c* oxidase subunit I (5’-COI) mitochondrial locus, which spans ca. 650 bp (PCR error rate = (650 × 0.10)/250 = 65/250). Albeit, this is probably a naïve estimate, as the total error rate depends highly on both the number of PCR cycles and the propensity for error in the polymerase employed, amongst other factors (Potapov and Ong 2017). Such a high PCR error rate is comparable in magnitude to Pacific Biosciences (PacBio) SEQUEL platform for Single Molecule Real Time (SMRT) sequencing, whose error rate of 13% for single basecalls in long reads up to 60 kb in length was noted by Hebert et al. (2018). However, as such errors tend to occur randomly, error rates are mitigated through continual sequencing of the same gene region via generation of a large number of circular consensus sequences (CCSs). Notwithstanding, Sanger sequencing is still considered the gold standard despite its high cost, with accuracies of 99.9%, often rivalling newer short read (< 400 bp) HTS machines with error rates of 0.8-1.7% (Hebert et al. 2018).

Screening high-volume DNA sequences for putative errors can reveal incorrect basecalls, chimeras/heteroplasmies, contaminants, insertion-deletion mutations (indels) and other nucleotide substitutions, as well as nuclear-mitochondrial (NUMT) insert/pseudogene amplification (Bandelt et al. 2001) within reference databases, such as GenBank (Harris 2003, https://www.ncbi.nlm.nih.gov/genbank) and the Barcode of Life Data Systems (BOLD, Ratnasingham and Hebert 2007, www.boldsystems.org). This is an important step for maintaining high levels of accuracy in assembled sequence records. Unlike in GenBank, which is not actively curated, users within BOLD currently can only flag questionable barcode sequences for subsequent examination (e.g. via specimen trace files) to ensure high sequence quality (Hanner 2009). However, it can be argued that neither database has been particularly successful in fully eliminating PCR, sequencing and other errors (Meiklejohn et al. 2019, Pentinsaari et al. 2020). Elimination of these errors (e.g. Stoeckle and Thaler (2014), Thaler and Stoeckle (2016)) is paramount to successful identification of specimens to species, phylogeographic haplotype analysis, studies of molecular evolution, characterisation of human diseases and the design of robust species primer/probe sets for forensic investigations.

DNA barcoding uses a small gene fragment from a standardised (orthologous) region of the genome to identify multicellular species (Hebert et al. 2003). In animals, this corresponds to a 648-658 bp fragment of 5’-COI (Hebert et al. 2003). As of December 2021, over 10.2 million DNA barcodes from animals, plants, fungi and protists have been catalogued within BOLD for almost 330000 species. With the number of specimen sequences in publicly accessible databases on the rise, it is crucial that their quality is not compromised. 

A key approach employed within many modern sequencing platforms to quantitatively assess putative errors stemming from incorrect nucleotide basecalls is the PHRED quality score (Ewing et al. 1998, Ewing and Green 1998). PHRED scores relate the probability of incorrectly calling a given base to the accuracy of said basecall on a logarithmic (base-10) scale. Higher PHRED scores indicate a lower probability of an incorrect basecall occurring and, thus, a greater overall accuracy in nucleotide assignment to electropherogram peaks. For instance, a PHRED score of 20 at a particular basepair position corresponds to an incorrect basecall probability of 0.01, meaning one error is expected to occur in every 100 sequenced nucleotides, resulting in a basecalling accuracy of 99%. The FASTQ file format incorporates both the nucleotide sequence for a particular read, along with position PHRED scores in ASCII format for easy portability. While PHRED scores offer an intuitive and simple way to measure sequencing integrity, a robust framework to easily visualise and quantify the impact of instrument errors from multiple sources in a DNA barcoding context is currently lacking. 

Stoeckle and Kerr (2012) first addressed the issue of sequencing errors within DNA barcodes using a frequency matrix approach, implemented in Microsoft Excel, to investigate the distribution of rare genomic variants (termed very low frequency variants (VLFs)) in a large avian dataset (11333 barcodes from 2706 species spanning 1038 genera and 149 families; 1-125 specimens/species; ca. 4.19 specimens/species). To do this, the occurrence of each positional nucleotide or amino acid in a set of DNA sequences was recorded in a data matrix. If a nucleotide/amino acid occurred at a frequency of < 0.001 (i.e. one error for every 1000 basepair positions), it was designated as a VLF and was noted as a potential sequencing artifact. Thus, a dataset consisting of at least 1000 taxon sequences is required to detect at least one true VLF. To further elucidate the precise origin of sequencing errors, VLFs were categorised as belonging to two distinct classes: singleton VLFs and shared VLFs. Singleton VLFs do not occur in other members of a species and tend to occur at the 5’ and 3’ ends of sequence reads; therefore, they are more likely to be errors in sequences, whereas shared VLFs are more consistent with known biological variation and tend to be randomly scattered throughout sequences. The distribution of singleton and shared VLFs within sequences can be explained by two primary factors arising during sequencing and assembly. Firstly, when viewing specimen trace files, sequence ends tend to be crowded and unevenly spaced, in addition to being often highly deteriorated with broad peaks that can be difficult to resolve (Athey 2013, Ewing et al. 1998, Ewing and Green 1998). As a result, misinterpretation of chromatograms and, thus, incorrect sequence editing, is common. Secondly, coverage is often lower at sequence ends (1×) compared to the middle (2×) from the forward and reverse primer (Athey 2013).

Here, we present *VLF *version 1.1 (Athey and McNicholas 2022, R Core Team 2022), an R package designed as a rapid and automated implementation of the method utilised by Stoeckle and Kerr (2012) to assess and indicate possible errors in DNA barcode sequences. DNA barcodes were of initial interest in this paper because of their broad application to specimen identification and because of their wide availability in online reference sequence databases. We validate the usefulness of the *VLF* package by first testing R functions on the avian barcode dataset of Stoeckle and Kerr (2012) and then applying the VLF pipeline in two ways: (1) to a subset of avian DNA barcodes comprising the Canada goose (*Branta canadensis*) and (2) to a newly-generated COI barcode dataset comprising sequence data from previously published studies related to seafood mislabelling of societally-important fish species. While we apply our method to DNA barcode data, such an approach is easily extended to other protein-coding sequence datasets well represented in online databases, such as those that make use of the mitochondrially-encoded cytochrome *b* (cyt*b*) gene. Further, while our focus is based solely on reads generated via Sanger-based amplicon sequencing, we stress that the approach outlined here could in theory also extend well to analysing DNA variation in Next-Generation Sequencing (NGS) and/or High-Throughput Sequencing (HTS) technologies, such as the PacBio SEQUEL platforms for downstream targeted environmental DNA (eDNA), metabarcoding, (mito)genome assembly or ancient DNA studies. 

## Implementation of the *VLF* package

**Figure 1.**
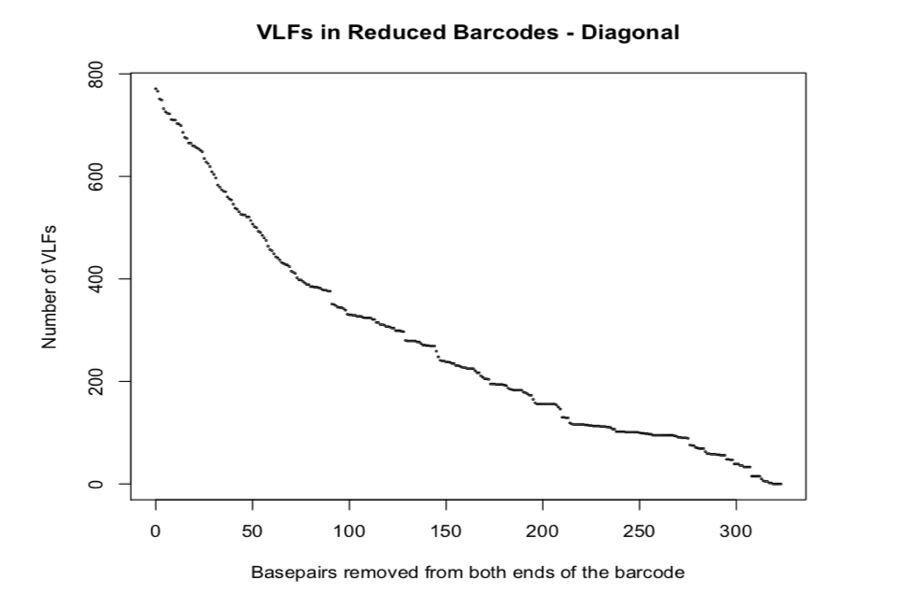
Plot depicting the effect of evenly reducing avian DNA barcode length at both 5’ and 3’ sequence ends on the overall presence of VLFs.

The *VLF* package inputs aligned DNA sequences as a matrix in FASTA format using the function fasta.read(file, seqlength = 648, pos1 = 1, pos2 = 3) and converts it into a sequence matrix. The first column of the matrix contains a specimen identifier, while the second gives the species name, followed by the DNA sequence in subsequent columns. The FASTA input header should be separated by ‘|’ and the 'pos1' and 'pos2' identifiers indicate the header’s position for the unique specimen identifier ('pos1') and the species name ('pos2'). For example, a FASTA header may be ‘>GBGC1668-06|NC 005317|Thunnus alalunga|COI-5P’, where GBGC1668-06 is the unique specimen identifier in the first position after the ‘>’ (pos1 = 1) and the species is *Thunnus alalunga* in the third position (pos2 = 3). The default sequence length is 648 bp. This function will automatically separate the FASTA file into a matrix containing the unique specimen identifier in the first column, the species name in the second column and the nucleotide sequence in the subsequent columns, one column per nucleotide. If the user wishes, they may also upload their sequences from their own format, provided the final sequence matrix follows these conventions. Sequence alignment can be handled using external software programmes such as MEGA (Kumar et al. 2016) to check whether indels are present within sequences and to verify that barcodes are in the correct reading frame when translated using the appropriate codon table. As well, *VLF *assumes that the first sequence position corresponds exactly to the first codon position. The 3' end of most primers is a first or second position, so it is rare that sequences trimmed to the primers will begin with a first codon position. Thus, users must exercise caution to ensure correct alignments prior to further analysis, especially if there is length variation within sequences to be assessed. VLF analysis with the *VLF *package may pose an issue for taxa that are known to harbour problematic artefacts within the barcode region, such as indels and NUMTs, derived from PCR or sequencing runs. Although indels and NUMTs/pseudogenes are rare in protein-coding genes such as COI, they are nevertheless common in various major invertebrate groups (including taxa such as Arachnida (Young and Hebert 2015), marine taxa (Schultz and Hebert 2022) and insects (Hebert et al. 2022)). Indels that do not occur in multiples of three (i.e. forming triplet codons) can lead to sequence frameshifts and, thus, alteration of overall protein function and their occurrence may be directly due to sequencing error or the presence of a NUMT/pseudogene. If a VLF leads to a change, not only in amino acid sequence, but also in the type of amino acid, this likely indicates a change in protein structure and may be a further indication of a potential error in barcode sequences (Athey 2013). Further, the presence of stop codons within sequence alignments due to a single-base indel can indicate the presence of NUMTs/pseudogenes which should be manually excluded by the user. Their presence can signal the premature termination of DNA translation if not eliminated naturally from species populations through purifying selection. If indels are found to be present within protein-coding sequence alignments from BOLD, the user should take several steps to deal with them. First, the associated specimen trace file(s) should be consulted and verified to be free of errors. This includes ensuring that both forward and reverse chromatograms are properly aligned with primers removed. Further, there should be no evidence of sequencing artefacts including heterozygous peaks, dye blobs, partial co-amplification, homopolymeric tracts or stop codons indicating possible reading frame shifts. Next, raw sequences should be realigned using altered parameters (e.g. gap penalties) or an alternative sequence alignment algorithm altogether, one that carries out both pairwise, in addition to, multiple sequence alignment (though at the cost of increased computation time) (e.g. ClustalW (Thompson et al. (1994)) instead of MUSCLE (Edgar 2004)). If indels are restricted to only one or a few sequences, the user may want to try to align sequences by eye, then verify that the resulting alignment is in the correct reading frame (i.e. free of stop codons) when translated to amino acids using the appropriate codon table. As a last resort, the user can simply exclude such sites or sequences entirely (e.g. if they are found to be associated with GenBank records). When scanning alignments for nucleotide VLFs (ntVLFs) and amino acid VLFs (aaVLFs), the user has the option of specifying a cut-off frequency (denoted *p*, not to be confused with* p*-value) different from the default of 0.001. The default value of *p* = 0.001 was selected because: (1) it was employed by Stoeckle and Kerr (2012) and (2) it resulted in a levelling off of singleton VLF occurrence to an asymptote as barcode length is reduced (while both shared and total VLFs showed an increasing linear trend; Fig. 1) (Athey 2013). The user must also specify a sequence length if different from the default 648 bp for nucleotides (or 216 residues for amino acids). Users can also analye a subset of sequences separate from reference sequences to allow easier interpretation of results and the elucidation of novel biological patterns within and between species using the function argument ‘own’ (see below for further explanation). For example, if there are 20,000 barcode sequences available for different species of fishes, but the user only has five sequences that they wish to assess, then the user can enter in the 20,000 barcode sequences as ‘x’ and their five sequences as ‘own’. In this way, a meaningful frequency matrix can be calculated and users can analyse their own sequences easily. 

The *VLF* package consists of three main functions: vlfFun(x, p = 0.001, seqlength = 648, own = NULL), aminoAcidFun(x, p = 0.001, seqlength = 216, own = NULL) and concordanceFun(nuc, aa, nuclength = 648, aalength = 216, aminoAcid.Modal). The functions vlfFun() and aminoAcidFun() have the same output: ‘modal’, ‘con100’, ‘conp’, ‘combine’, ‘specimen’, ‘position’, ‘sas’ and ‘VLFmatrix’. The ‘modal’ object contains the sequence of nucleotides or amino acids that occur most often in each position, based on the calculated frequencies. The ‘con100’ value gives the number of sequence positions that are 100% conserved amongst all specimens in the dataset, while the ‘conp’ value gives the number of sequence positions that are (1 - *p*)% conserved (i.e. if using the default value of *p *= 0.001, then (1 - *p*)% = 99.9%). The ‘combine’ value gives the number of amino acid positions that are (1 - *p*)% conserved for the first and second modal sequence (i.e. the two most common sequence variants in a taxon dataset). ‘Specimen’ is a vector containing the number of VLFs for each specimen in the dataset and 'position' is the number of VLFs for each sequence position in the dataset. The value ‘sas’ gives the number of singleton and shared VLFs in each sequence position of the dataset. Lastly, ‘VLFmatrix’ is a reduced matrix containing only VLFs, with “NA”s in any position that does not contain a VLF. Additionally, if the user specifies their own sequences, then the programme outputs specimen VLF counts (‘ownSpecCount’), position VLF counts (‘ownPosCount’), a VLF matrix containing all “own“ specimens (‘ownVLFMatrix’) and a reduced VLF matrix containing only those specimens which have VLFs in their sequence (‘ownVLFreduced’). This output allows the user to assess their own sequences of interest more easily, without having to filter through large datasets. The third main function of the *VLF* package is concordanceFun(nuc, aa, nuclength = 648, aalength = 216, aminoAcid Modal), where ‘nuc’ and ‘aa’ are the VLFmatrix outputs of the vlfFun() and aminoAcidFun() functions, respectively, ‘nuclength’ and ‘aalength’ are the sequence lengths for the nucleotide and amino acid sequences, respectively (648 bp and 216 residues by default) and ‘aminAcidModal’ is the modal output of aminoAcidFun(). The main goal of the concordanceFun() function is to calculate how many nucleotide VLFs occur within the codon of an amino acid VLF. The output for this function is a list of concordant nucleotide and amino acid VLFs ('matched'), a calculation of how many concordant VLFs there are for each codon position ('codons'), the number of concordant amino acid VLFs that changed amino acid residue type (‘concordantType’), the number of overall amino acid VLFs that changed amino acid residue type (‘aminoAcidType’), the overall number of nucleotide VLFs and amino acid VLFs that showed concordance (‘concordNuc’ and ‘concordAA’, respectively) and the number of sequences that contained both nucleotide VLFs and amino acid VLFs (‘sequences’). 

The *VLF* package also has several other useful functions, such as one to calculate singleton, shared and total VLF error rates, based on a high degree of conservation at second codon positions (Error.Rate(single, shared, spec, seqlength)). In computing total error rates, both singleton and shared VLFs should be considered. This is because, despite shared VLFs making up a negligible fraction of overall sequences, they comprise a high proportion of sequences with VLFs (Athey 2013). However, this was not done by Stoeckle and Kerr (2012), who only calculated an overall singleton error rate. As such, we introduce modified formulae for the calculation of putative VLF error rates (ERs) as follows:

\begin{varwidth}{50in}\begin{equation*}
            \mathrm{Singleton \; ER} = \mathrm{\frac{\mathrm{2nd \; Position \; Singleton \; VLFs}}{\Big(\frac{2nd \; Positions}{Barcode}\hspace{1mm} - \hspace{1mm}2nd \; Position \; Shared \; VLFs\Big)\Big(Number \; of \; Barcodes\Big)}}
        \end{equation*}\end{varwidth}

\begin{varwidth}{50in}\begin{equation*}
            \mathrm{Shared \; ER} = \mathrm{\frac{\mathrm{2nd \; Position \; Shared \; VLFs}}{\Big(\frac{2nd \; Positions}{Barcode}\hspace{1mm} - \hspace{1mm}2nd \; Position \; Singleton \; VLFs\Big)\Big(Number \; of \; Barcodes\Big)}}
        \end{equation*}\end{varwidth}

\begin{varwidth}{50in}\begin{equation*}
            \mathrm{Total \; ER} = \mathrm{\frac{\mathrm{2nd \; Position \; VLFs}}{\Big(\frac{2nd \; Positions}{Barcode}\Big)\Big(Number \; of \; Barcodes\Big)}}
        \end{equation*}\end{varwidth}

A useful feature of the *VLF* package is the ability to distinguish VLFs that are shared between members of the same species (i.e. occurring in two or more sequences) or that are singletons (i.e. occurring in only a single individual). In the case of singleton sequences, it is important to know how they manifest in large barcode libraries. There are two possibilities: (1) only a single specimen of a species was sampled or (2) multiple individuals within a species lacking true genetic polymorphisms were sampled (Talavera et al. 2013). This information can be used to assess whether VLFs arise as a result of sequencing error or divergence, since with small sample sizes, actual biological variants (i.e. true haplotypes) may be misidentified as VLFs; whereas, very heavily sampled species will have a higher incidence of their biological variants (Athey 2013). In utilising DNA barcodes for biodiversity or evolutionary studies, the presence of one or two VLFs (equivalent to 0.15-0.30% K2P (Kimura Two Parameter; Kimura (1980) distance) is not likely to hinder specimen assignment as the majority of species will differ by > 2% in their barcodes (Hebert et al. 2003, Hebert et al. 2003, Stoeckle and Kerr 2012). Since VLF occurrence is expected to be low within taxon records, a VLF is not likely to cause a barcode sequence to appear more closely related in distance to a distinct species (Athey 2013). This is the case for species displaying many VLFs, as a VLF will result in a given specimen becoming equally distant from all others in a taxonomic group (Athey 2013). However, when DNA barcodes are used in the design of molecular assays for accurate species detection of potentially mislabelled seafood products (e.g. primer/probe synthesis), the presence of even a single nucleotide difference can greatly inflate the number of false positive and false negative errors. In such cases, alternative methods of species identification, apart from traditional distance-based approaches, are often employed (e.g. diagnostic nucleotides; Sarkar et al. (2008), Wong et al. (2009)). Thus, VLF analysis is expected to be well utilised within socioeconomic contexts. In such cases, it is imperative that a high level of species sequence identity be achieved (often ca. 98% for instance, but ideally a 100% query match to a reference in the library is needed). The *VLF* package can aid in this endeavour by eliminating questionable sequences having a high incidence of VLFs, including only those DNA sequences with a low proportion of VLFs.

In a study by Phillips et al. (2015) utilising DNA barcodes from ray-finned fishes (Chordata, Actinopterygii), it was found that the random sampling of hundreds to thousands of individuals per species will likely be required to uncover the majority of estimated haplotype variation within a given species. In the case of Actinopterygii, which is a group that is known to possess high levels of intraspecific genetic diversity, it seems plausible that much of the biological variation seen within and between species actually comprises spurious or non-unique (i.e. duplicate) haplotypes (Hickerson et al. 2006, Dasmahapatra et al. 2010, Fietz et al. 2013). Phillips et al. 2019 highlighted the strong relationship between VLFs and required specimen sample sizes: higher sampling coverage means true haplotype variation will be less likely flagged as VLFs. A large proportion of COI DNA barcodes within BOLD are mined from GenBank. Unfortunately, such records often lack appropriate metadata requirements necessary for compliance with BARCODE standards set out by the Consortium for the Barcode of Life (CBOL) (Hanner 2009). This was the primary reason for excluding GenBank records in Phillips et al. (2015)'s study, despite resulting in lower initial sample sizes on which to probe current levels of sampling effort for fishes.

The question, therefore, that must be addressed is: does there exist an optimal threshold size for specimen sampling above which no new genetic (i.e. DNA barcode haplotype) variation is likely to be observed for a species? That is, can all (or nearly all) DNA barcode haplotype diversity for a species be uncovered by simply sampling *N *individuals? If so, how confident can one be in such an estimate? Phillips et al. (2015), Phillips et al. (2019) and Phillips et al. (2020) term this *sampling sufficiency*, which is defined as the sample size at which sampling accuracy is maximised (or converged) and above which no new sampling information (i.e. DNA barcode haplotype variation) is likely to be gained. However, caution is required in adopting this definition since exhaustively sampling taxa of interest may result in only small gains in accuracy (Phillips et al., in preparation). Despite this caveat, if such a lower bound estimate exists, it would provide a useful stopping criterion for specimen sampling since it is the best guess presently available (Phillips et al. 2015, Phillips et al. 2019, Phillips et al. 2020). Future work should, therefore, employ the R package *HACSim *(Phillips et al. 2020), which will ensure a representative sample of COI variation, to assemble representative taxon BARCODE datasets, based on BOLD or GenBank specimen records for direct assessment of VLFs using the *VLF* package.

It is well known that current sample sizes within barcode libraries are likely insufficient for making inferences at the phylogenetic level, for instance, in the calculation of divergence times of sister taxa via neutral coalescent/molecular clock models, but there is evidence that suggests otherwise (e.g. Lavinia et al. (2016)). Early on, the DNA barcode gene region was believed to be too short to aid in reliable tree reconstruction due to relatively low phylogenetic signal since multiple genetic markers must often be considered to conclusively yield meaningful information on the evolutionary history of a single taxon (Hajibabaei et al. 2007). However, because phylogenetically-informative mitochondrial loci, with the exception of COI (and to a lesser extent cyt*b*), are available for only a handful of taxa within global sequence databases, phylogenetic interpretations can become obscured (Wilson 2011). Despite this, neighbour-joining trees are routinely used in DNA barcoding studies as an identification tool to flag sequences originating from potential contaminants (e.g. bacterial symbionts like *Wolbachia *(Smith et al. 2012)) or to pinpoint sequences that may reflect non-functional gene copies (i.e. NUMTs/pseudogenes), both of which may be complicated by mitochondrial introgression. The impact of sampling on the presence of VLFs in taxon sequence records is an important consideration in the assessment of overall sequence quality within barcode libraries; however, questions still remain concerning optimal sample sizes required for such assessments.

The *VLF* package also contains functions to give visual outputs of the distribution of VLFs throughout the sequences. Decile.Plot(VLF, seqlength = 648) creates a decile plot showing the number of VLFs in every tenth of the sequence. The input ‘VLF’ is the ‘position’ output of the vlfFun() and aminoAcidFun() functions, containing the counts of VLFs in each position of the sequence. The user may also enter in the ‘sas’ output of these functions, to create a decile plot of both the single and shared VLFs. Similarly, the *VLF* package also contains the function Sliding.Window(VLF, seqlength = 648, n = 30) which creates a sliding window plot of VLFs with a default window size of 30 bp. A 30 bp *k*-mer window was selected by Stoeckle and Kerr (2012) to eliminate as much noise in the data as possible while clearly showing the precise distribution of singleton and shared VLFs within barcode sequences. Sliding windows are useful for this type of analysis because they offer a glimpse into how the number of observed VLFs change as the window is shifted along the barcode segment from the 5’ to 3’ end by a fixed amount (one basepair by convention) in the fashion of a moving average. Such plots have been used within the DNA barcoding literature to select informative minibarcodes for optimal specimen identification in taxa such as earthworms, using sequencing technologies like pyrosequencing (Boyer et al. 2012).

## Results

In the following subsections, focus is placed specifically on ntVLFs (hereafter simply referred to as VLFs) for the sake of brevity. Required DNA sequence data is included in Suppl. material 1. Code to reproduce all analyses can be found in Suppl. material 2.

### Application of the *VLF* package to avian DNA barcodes

Aligned avian barcode sequences, identified to at least the family level, were downloaded from the supplementary material of Stoeckle and Kerr (2012) in FASTA format. Birds were the taxon of choice because they are amongst the best-represented groups within barcode libraries, have well-defined species boundaries, as well as large and well-documented census population sizes (Stoeckle and Kerr 2012, Stoeckle and Thaler 2014). These sequences were initially retrieved from GenBank using the keyword ‘BARCODE’ (Hanner 2009), which ensures sequences are at least 500 bp in length, contain less that 1% ambiguous bases (Ns) and have associated trace files and primers within BOLD, amongst other requirements and optional metadata (such as specimen images and GPS coordinates). The birds nucleotide dataset can be accessed using the R code data(birds); the amino acid dataset can be accessed by using the R code data(birds_aminoAcids). 

**Figure 2.**
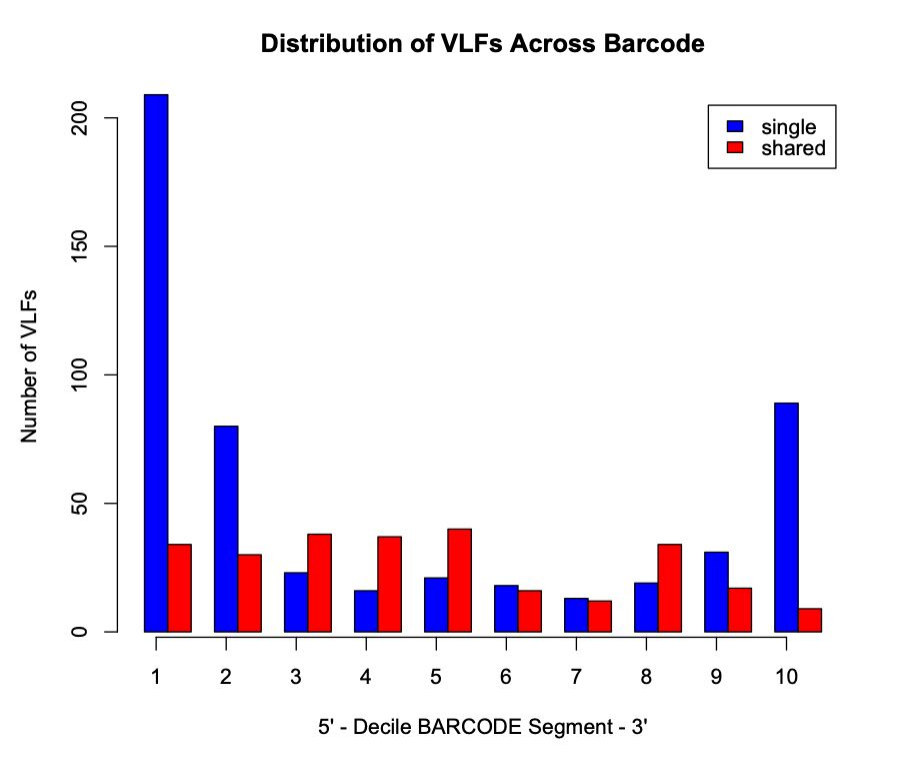
Decile plot showing the distribution of singleton (blue) and shared (red) VLFs across the barcode segment in avian barcodes.

**Figure 3.**
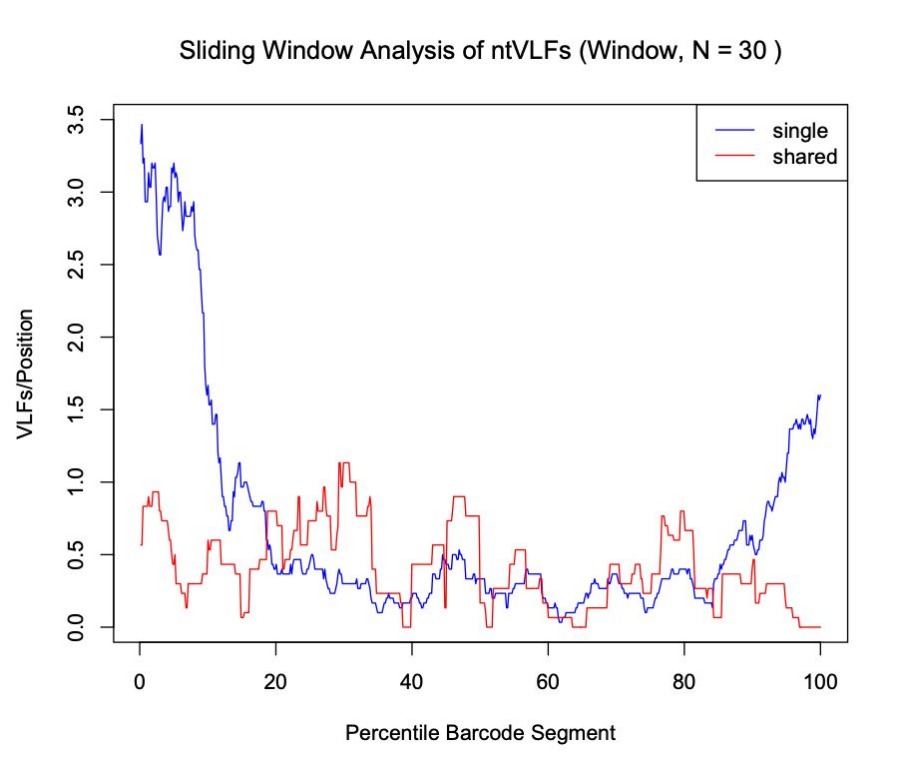
Sliding window plot depicting the distribution of singleton (blue) and shared (red) VLFs in avian barcodes. A default window size of 30 nucleotides was selected to minimise stochasticity apparent in the data.

Sequences were then analysed in R using the three primary functions of the *VLF* package outlined above in conjunction with others. Results were concordant with those of Stoeckle and Kerr (2012) (Fig. 2, Fig. 3). Reproducing the full analysis of Stoeckle and Kerr (2012)'s dataset using the *VLF* package gave nearly identical results, but took less than one minute (6.723 s) using vlfFun() on a Mac OS X 11.4 machine (2.7 GHz Dual-Core Intel Core i5 processor, 8 GB 1867 MHz DDR3 memory), while the conventional analysis, using an Excel spreadsheet, took several days (actual numbers unknown since this is dependent on memory used by macros). 

In comparing results obtained via *VLF *to those found by Stoeckle and Kerr (2012) Excel implementation, two discrepancies are noteworthy. The first relates to the occurrence of synonymous species names. Stoeckle and Kerr (2012) found a total of 573 singletons within the avian dataset, whereas in employing R, 582 singletons were observed by Athey (2013). This difference is likely because the present study simply checked for species names only occurring once, without accounting for any prior taxonomic knowledge. Secondly, a total of 768 specimen VLFs (494 singleton VLFs, 274 shared VLFs) from 549 barcodes were noted by Stoeckle and Kerr (2012) when singleton and shared VLFs were pooled together, in comparison to findings herein, where 771 specimen VLFs (between 1-15 VLFs for each specimen) and 771 positional VLFs (510 singleton VLFs, 261 shared VLFs, between 1-18 VLFs for each position) were observed across 552 sequences and 241 sequence positions, respectively. A singleton (gi|359282265|gb|JQ174997.1, 651 bp), corresponding to the species *Halcyon smyrnenis* (White-throated kingfisher) possessed the most VLFs. Alignment position 308 comprised the most VLFs. Using *VLF*, the distribution of specimen and positional VLFs was easily determined (Table 1 and Table 2). Singleton, shared and total error rates, computed using the function Error.Rate(), are given in Table 3. While the *VLF* package automatically compared species names for counts of singleton and shared VLFs, Stoeckle and Kerr (2012) manually separated and compared VLFs. Thus, it is possible that Stoeckle and Kerr (2012) counted some sequences that contained both shared and singleton VLFs as only shared VLFs, or vice versa, which may account for the observed decrease in VLF count. The small difference in sequence count is not accounted for, but has negligible effect on the overall results. As *VLF* does not require manual assessment and because of the speed of the computation, the* VLF* package is the most appropriate available tool for a large-scale VLF analysis. 

Another advantage of the *VLF* package is automation of the analysis. To perform this analysis using Excel, the user must manually enter macros for each individual dataset. The automation of the analysis makes it a user-friendly tool that can be utilised as a clean-up step during a barcode analysis workflow.

**Figure 4.**
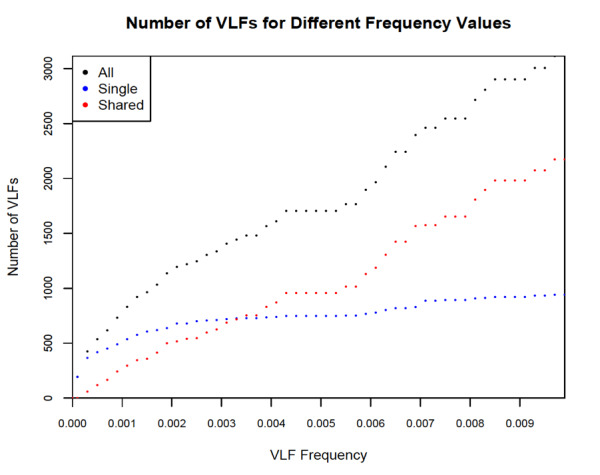
Plot depicting choice of VLF frequency on the number of observed single, shared and total VLFs across avian barcodes.

**Figure 5.**
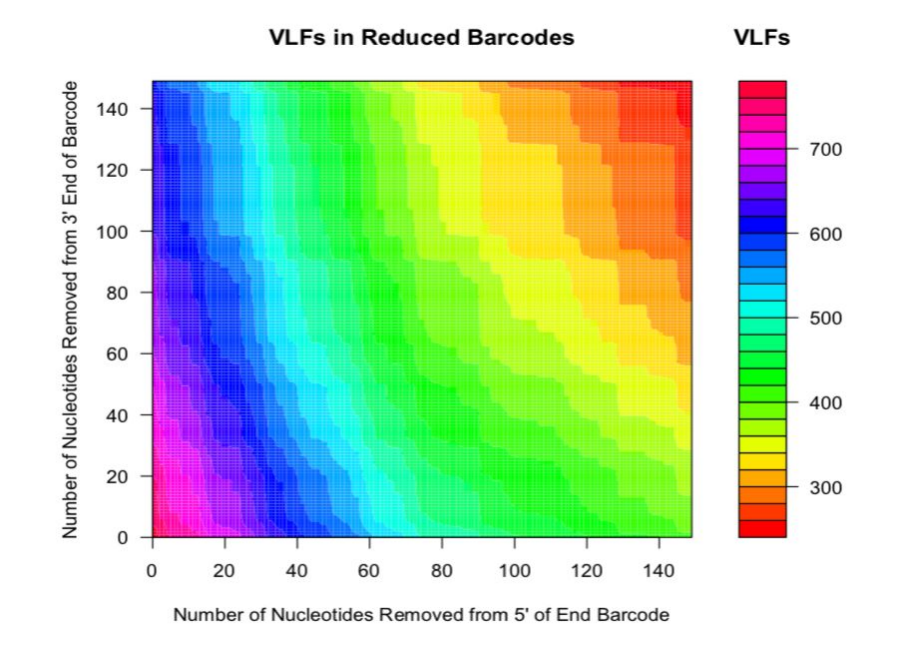
Contour plot displaying the effect of evenly shortening sequences by fixed amounts from the 5’ end to reduce overall numbers of VLFs across avian barcodes.

In addition, the effect of reducing full-length avian barcodes evenly at both the 5’ and 3’ ends and the choice of VLF frequency cut-off, on the presence of VLFs is clearly illustrated in Fig. 4 and Fig. 5, respectively. The former figure depicts a contour heatmap plot of the total number of VLFs observed as a result of shortening barcodes on both 5’ and 3’ sequence ends. In that image, deeper colour intensities associated with higher overall numbers of VLFs within sequences, are directly proportional to the number of nucleotide bases removed. Such a plot represents a novel way of examining DNA sequences for the presence of machine errors (in conjunction with sliding windows and decile plots presented herein, as well as in Stoeckle and Kerr (2012)'s original study).

### Probing species-specific VLFs in avian DNA barcodes

Taxa with large numbers of collected specimens should be expected to show strong VLF signals relative to the real biological variation present in DNA sequences. Thus, in addition to investigating the prevalence of VLFs at the class level (Aves), the incidence of VLFs at the species level was assessed for *Branta canadensis* (Canada goose), the species with the largest number of specimens (125) in Stoeckle and Kerr (2012)'s dataset. The Canada goose is widely known as a nuisance species that has become well-adapted to urban human environments. This species was noted as a strong outlier in comparison to other taxa by Stoeckle and Kerr (2012). Analysis of this and other species in the birds' dataset is easily accomplished by first using the separate() function in *VLF*, which rapidly partitions specimen records into lists according to species name, followed by passing the reduced dataset to the ‘own’ argument to vlfFun() (or another function that takes the same argument). *B. canadensis *corresponded to list element 317 upon applying the function. While more than 100 conspecifics were found to lack VLFs for this species, closer examination of specimen trace files revealed the presence of double peaks at VLF sites (Stoeckle and Kerr 2012). Such a pattern is highly suggestive of co-amplification of a short pseudogene at the 5’ end of examined barcodes.

**Figure 6.**
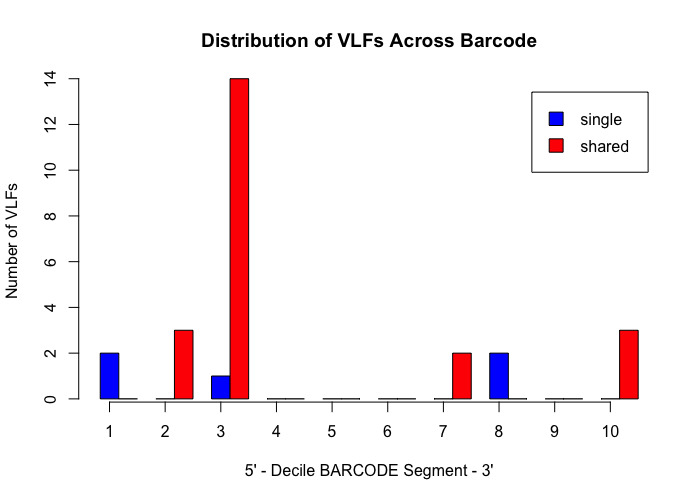
Decile plot showing the distribution of singleton (blue) and shared (red) VLFs across the barcode segment in Canada goose (*Branta canadensis*) barcodes.

**Figure 7.**
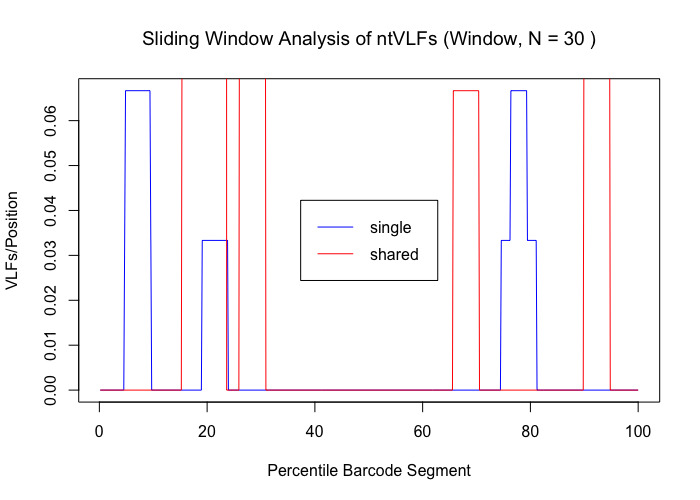
Sliding window plot depicting the distribution of singleton (blue) and shared (red) VLFs in Canada goose (*Branta canadensis*) barcodes. A default window size of 30 nucleotides was selected to minimise stochasticity apparent in the data.

Analysis of the Canada goose dataset revealed a total of 27 specimen VLFs (between 1 and 3 VLFs for each specimen) across all 125 examined barcode sequences (18 specimens comprised VLFs). Similarly, 27 positional VLFs were observed across the entire 648 bp barcode segment (five singleton VLFs, 22 shared VLFs, between 1-10 VLFs for each position). Ten alignment positions displayed VLFs: sites 58, 59, 124, 145, 147, 190, 435, 490, 501, 535. Position 145 contained the most VLFs at 10, while all other sites had between 1 and 4 VLFs. Most VLFs were concentrated at the 5’ end of sequences, with 15 VLFs occurring within the third decile alone (Fig. 6). All other deciles had between 2 and 3 VLFs. Within the sliding window, the highest positional VLF error rate (ca. 0.5 VLFs) occurred near the 20^th^ percentile (Fig. 7). Specimen and position VLF distributions are given in Table 4 and Table 5, respectively. Calculated error rates are found in Table 6.

### Application of the *VLF* package to DNA barcoding forensics

Seafood fraud is a growing economic and ecological problem facing society today. DNA-based identification of specimens to species (e.g. DNA barcoding) is increasingly being used as a means of verifying product integrity. The availability of such technologies is important given that species of higher economic value (e.g. halibut, red snapper) are often substituted with those of lower cost (e.g. catfish, tilapia) (Hanner et al. 2011a, Naaum and Hanner 2015). Thus, it is imperative that new tools be developed to aid governmental regulatory agencies, such as the Canadian Food Inspection Agency (CFIA) and the United States Food and Drug Administration (USFDA) in combatting this mounting issue. VLF analysis represents one potential solution in this respect.

To assess the utility of *VLF *to the field of barcoding forensics for regulatory purposes, DNA barcodes from four research studies published between 2008 and 2011 (Wong and Hanner 2008, Rasmussen et al. 2009, Wong et al. 2009, Hanner et al. 2011b) were downloaded from the BOLD4 database on 30 November 2016 using the BOLD project codes ‘EMRKT’ (Fish Market Survey), ‘SSNA’ (Salmonid Species North America), ‘EWSHK’ (Shark Barcoding Using a Nucleotide Diagnostic Approach) and ‘EBFSF’ (Billfish and Swordfish COI Identification), respectively. EMRKT comprised a single Echinodermata sequence (EMRKT065-07, *Mesocentrotus franciscanus* (Red sea urchin), 633 bp with Ns excluded from the 3’ end), which was treated separately from the fish barcodes. The final dataset consisted of 2371 barcode sequences from 44 genera, 72 families and 114 species (ca. 20.80 specimens/species; Table 7). Only EMRKT and EBFSF were comprised partially of barcodes > 500 bp in length. Barcodes shorter than this cut-off were nevertheless, infrequent in EWSHK and SSNA projects and were not removed prior to VLF analysis.

Sequence alignment was carried out in MEGA6 using MUSCLE and the ‘Align DNA’ option with default parameters. Ends of the aligned sequences were then trimmed to the standard barcode length for fishes (i.e. 652 bp) and subsequently translated to amino acids using the ‘Vertebrate Mitochondrial’ and the ‘Invertebrate Mitochondrial’ codon tables. Alignments were checked for the absence of stop codons and verification that they were in the correct reading frame. Sequencing artifacts were common within DNA barcodes. For example, a single-base indel (specifically, a nucleotide deletion), identified using the SequenceMatrix (Vaidya et al. 2011) tool within the TaxonDNA software (Meier et al. 2006, Vaidya et al. 2011), was present in one specimen from the SSNA BOLD project for *Oncorhynchus keta* (Chum salmon, SSNA943-08, 606 bp, position 367) and, while presumed to be the result of sequencing error, was not excluded from analysis since the intent here is to demonstrate that such errors are evident and persist in reference DNA sequence libraries.

**Figure 8.**
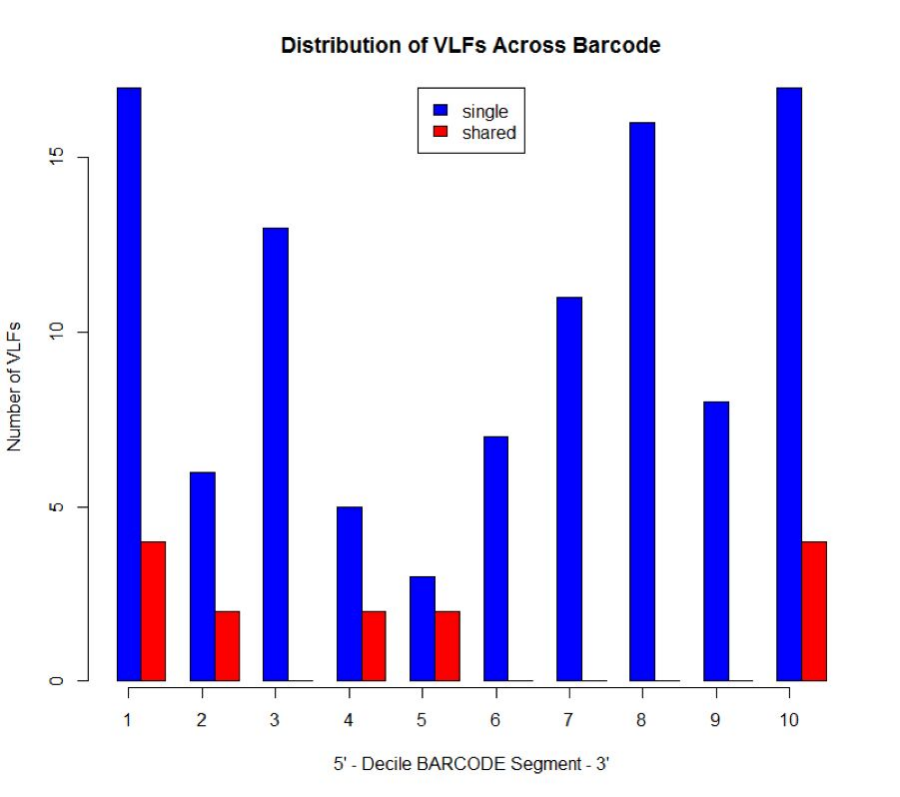
Decile plot showing the distribution of singleton (blue) and shared (red) VLFs across the barcode segment in fish barcodes.

**Figure 9.**
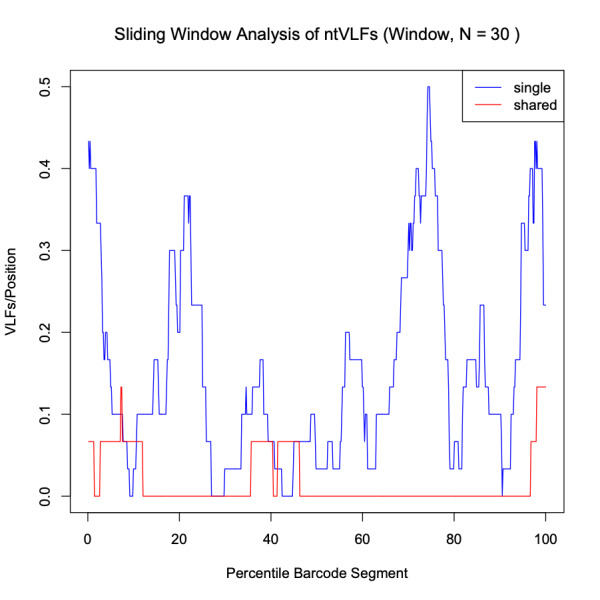
Sliding window plot depicting the distribution of singleton (blue) and shared (red) VLFs in fish barcodes. A default window size of 30 nucleotides was selected to minimise stochasticity apparent in the data.

Findings are presented below (Fig. 8, Fig. 9). A total of 117 specimen VLFs were detected (between 1 and34 VLFs for each specimen) across all 2371 COI sequences (58 specimens displayed VLFs). Similarly, 117 positional VLFs were noted (103 singleton VLFs, 14 shared VLFs, between 1 and 3 VLFs at each position) across the entire barcode region. VLFs were identified at 84 alignment sites. Positions 155, 618, 636 and 639 comprised the most VLFs. While singleton VLFs were otherwise uniformly frequent across the barcode region, they were lowest in the middle (within the fifth decile and 50^th^ percentile). The distribution of specimen and positional VLFs is shown in Table 8 and Table 9. Computed error rates can be found in Table 10. Error rates were similar in magnitude across all datasets examined herein and to that of Stoeckle and Kerr (2012) who calculated a singleton error rate of ca. 8.04 x 10^-5 ^errors/bp (8.04 x 10^-5 ^errors/bp x 648 bp/barcode = ca. 0.05 errors/barcode), as well as Athey (2013) who found ca. 8.54 x 10^-5 ^errors/bp (ca. 0.06 errors/barcode). These results are strong evidence for high sequence quality of published and unpublished taxon records mined from GenBank and BOLD. 

## Discussion

Stoeckle and Kerr (2012) were the first to address the issue of DNA barcoding errors using a frequency matrix approach. Their analysis showed that singleton VLFs occur more frequently at the 5’ and 3’ ends of sequence reads, making them more likely to be errors in sequences. Based on this observation, it stands to reason that trimming full length (ca. 650 bp) barcode alignments by ca. 50 bp (ca. 25 bp on both sequence ends) down to ca. 600 bp should reduce much of the existing VLFs and, thus, also overall error rates. However, this trimming figure is arbitrary and will likely depend on a number of factors including the taxonomic group under investigation, the choice of primers employed for sequence amplification (e.g. universal, specific or cocktail) and the choice of molecular gene marker. The 5’ end of sequences is known to be considerably noisier than the 3’ end (Stoeckle and Kerr 2012), owing to greater difficulties during targeted amplification and sequencing (Ivanova et al. 2007). Thus, researchers should consider multiple different trimming thresholds when conducting their own analyses. Barcode length is expected to affect the number of haplotypes observed for a species, which is evident in fungi, for example (Min and Hickey 2007). Short sequences that are shared between two species are presumed to be evolutionarily older, while longer sequences have a more recent origin (Racimo et al. 2015). Shortening sequences may remove important biological information; however, this strategy would likely not hinder species-level assignment, as various studies have aptly demonstrated that barcodes as short as 200 bp can still lead to correct taxon identification of an unknown degraded animal sample with upwards of 90% accuracy (Hajibabaei et al. 2006, Meusnier et al. 2008). However, artifacts such as NUMTs/pseudogenes are less easily detected in short reads (Porter and Hajibabaei 2021). Thus, novel computational and statistical approaches are needed to better uncover machine errors and artifacts within Sanger-derived DNA sequence libraries housed in large genomic repositories. 

Since the publication of Stoeckle and Kerr (2012)'s study, VLF analysis has not been widely utilised as an alternative method (e.g. compared to PHRED scores in sequence trace files) to evaluate the quality of DNA sequences available in online libraries, such as GenBank and BOLD**. **A brief literature search (as of December 2021) revealed only 20 peer-reviewed publications that explicitly mention Stoeckle and Kerr (2012)'s work. Of these, only a handful adopt Stoeckle and Kerr (2012)'s trimming approach to minimise barcode errors. For instance, Stoeckle and Thaler (2014) trimmed full-length (648 bp) avian barcodes by 10% (ca. 65 bp) on both the 5’ and 3’ ends (down to 519 bp) to reduce the overall contribution of (singleton) VLFs. Other studies have followed a similar path (Stoeckle and Coffran 2013, Chakraborty and Ghosh 2015, Collins et al. 2015, Chakraborty et al. 2017, Machado et al. 2018, Sanchez-Velasquez et al. 2021).

The *VLF* package is a useful tool for assessing errors in DNA sequences; however, the presence of a single VLF is not always an indication of biological error and so caution must be exercised when investigating these cases. When VLFs occur, it is advisable to assess whether they are singletons or shared between multiple specimens/species. The specific analyses carried out herein suggest that singleton and shared VLFs may occur outside the narrow 3’ and 5’ windows as seen in Fig. 2, Fig. 3, Fig. 6, Fig. 7, Fig. 8, and Fig. 9. For example, Fig. 9 indicates that the highest incidence of shared VLFs for fishes occurs in the 70^th^-80^th^ percentile of the barcode segment, as opposed to the sequence ends. This could be due to the relatively low sample size of the examined dataset overall (despite a high number of specimens per species on average) when compared to the much larger birds dataset, meaning that true biological variation has potentially been misconstrued as PCR/sequencing error. Further, the figures suggest that shared VLFs are more prevalent within 5’ and 3’ windows rather than outside. Thus, researchers must carefully exercise vigilance when trying to distinguish errors from actual haplotype variation. If VLFs are shared between members of the same species, then examination of morphological traits, geographic/ecological range and evolutionary history of those specimens sharing a VLF may be of interest to determine if these VLFs are new biological variants in individuals separated from other members of the same species. When VLFs are detected, it is recommended that the original trace file be examined to determine if an incorrect basecall is present. This may help curate sequence databases for the further application of the DNA sequences. If multiple VLFs occur within a single specimen, then this may be an indication of a NUMT/pseudogene or a chimeric sequence. This explanation seems plausible for *M. franciscanus*, whose record showed a high degree of sequence noise (34 VLFs). Interestingly, Wong and Hanner (2008) found that this record returned three conflicting species matches to three distinct genera with sequence similarities below 90% in both the BOLD ID Engine and BLAST (https://blast.ncbi.nlm.nih.gov/Blast.cgi; Altschul et al. 1990) and showed a K2P minimum interspecific distance of 34.48% (nearest neighbour: EMRKT013-07, *Elagatis bipinnulata *(Rainbow runner)) using the DNA Barcode Gap Analysis tool within the BOLD Workbench, suggesting that VLFs may indeed pose significant obstacles for specimen discrimination, contrary to previous expectations. If no ambiguous basecalls are detected, VLFs may be the result of biological variants. 

Note that this method is only useful with large datasets of sequences since the default cut-off frequency for VLF designation is *p* = 0.001. Therefore, a dataset with at least 1,000 sequences is required, but even larger datasets are suggested. Having as much haplotype variation as possible for a given taxon is ideal; however, the datasets should not be so deeply divergent that many specimens are expected to have vastly different sequences from other specimens within the same dataset. As well, the datasets should contain multiple members from each species, to ensure adequate representation of singleton and shared VLFs. It is suggested to use 5-10 individuals per species, if possible, which is typical for most barcoding initiatives conducted to date (Phillips et al. 2015, Phillips et al. 2019, Phillips et al. 2020, Phillips et al. 2022), but smaller numbers (e.g. 1 or 2 sequences), which may arise in the case of rare taxa, restricted geographic sampling or project costs/funding, may also be acceptable. In these scenarios, caution must be exercised when interpreting results as findings will likely be biased at low sample sizes. In contrast, Phillips et al. (2015) found that between 150 and5400 individuals per species must be collected to uncover all estimated haplotype variation for species of Actinopterygii using a crude sampling model, based on uniformity of species’ haplotypes. That approach served as a canvas from which to develop more sophisticated methods. To this end, novel computational tools, such as *HACSim* should be employed to assess likely required specimen sample sizes for well-inventoried species of interest. This improved method over that of Phillips et al. (2015) makes use of species’ haplotype frequency distributions to iteratively propose improving estimates of sampling sufficiency, based on an initial guess and provided haplotype diversity recovery thresholds, along with saturation levels observed in haplotype accumulation curves. For instance, using a non-parametric stochastic statistical resampling scheme, *HACSim *predicts that sample sizes of 414, 604 and 803 individuals for scalloped hammerhead shark (*Sphyrna lewini*), lake whitefish (*Coregonus clupeaformis*) and deer tick (*Ixodes scapularis*), respectively, based on initial estimates of 171, 235 and 349 specimens represented in DNA sequence alignments, are likely required to capture at least 95% of 5’-COI haplotype variation observed for these species. Having sufficient sample sizes allowing broad representation of real taxon-level genetic diversity is critical for enabling reliable detection of taxon barcode gaps with high statistical power and confidence when they actually exist (Meyer and Paulay 2005, Phillips et al. 2022). VLF analysis appears to be a promising avenue to explore in this regard.

It is important to consider the ways in which VLF assessment may be implemented into the BOLD system, as biological variants should not be tagged as sequence errors. An interesting, but noteworthy connection exists between the occurrence of sequencing errors within barcode records and the Barcode Index Number (BIN) framework (Ratnasingham and Hebert 2013). As specimens assigned to operational taxonomic units (OTUs) closely mirror actual species, the *VLF* R package can be directly utilised to detect artificial biological variants that may be missed by other assessments. Four levels of BIN assignment are possible: MATCH, SPLIT, MERGE or MIXTURE. Only BIN MATCHES are concordant with current Linnean taxonomy. A BIN SPLIT, in which sequences fall into two or more OTUs (i.e. erroneous lumping of named species), indicates potential cryptic species diversity; whereas, BIN MERGES and MIXTURES suggest premature splitting of named species or cases of species synonymy and specimen misidentification or species hybridisation, respectively (Ratnasingham and Hebert 2013, Serrao et al. 2014). Although BINs are inherently dynamic, a stand-alone BIN (i.e. a BIN MATCH) containing only one specimen may indicate that the sequence is erroneous; however, it may also indicate a lack of reference barcodes. Thus, VLF analysis can be integrated with the BIN framework to identify poor quality sequences containing VLFs, standalone BINs with VLFs or a singleton VLF found within a large BIN.

VLF analysis is a useful tool for evaluating errors in sequence records. The *VLF* package allows users to quickly and easily assess their own barcode records without the need for manual configuration or the use of Excel. While we tested the *VLF* R package on a previously-studied avian barcode dataset, as well as investigated the distribution of VLF sequencing errors in DNA barcodes from a variety of seafood species to probe the incidence of product mislabelling, we suggest the programme be used further to assess sequence errors in other large BARCODE and non-BARCODE libraries within GenBank and BOLD, such as Lepidoptera. Inspection of species-specific sliding window plots could indicate highly-variable nucleotide sequence regions subject to high mutation rates (such as that indicated by the sharp peaks in Fig. 7) and, thus, strong levels of selection (e.g. selective sweeps) acting on species populations. Definitive evidence of the impact of NUMTs/pseudogenes could be easily checked through either the computation of GC content, inspection of open reading frame (ORF) length or the calculation of per site non-synonymous to synonymous substitution ratios (dN/dS; Porter and Hajibabaei 2021). Both GC content and ORF length have been found to be lower/shorter in NUMTs/pseudogenes when compared to true haplotype variants. dN/dS fractions close to one are indicative of the presence of non-functional gene copies; conversely, ratios much less than one are expected for functional genes since substitutions primarily occur within non-synonymous sites, thus preserving overall amino acid composition and structure, which is crucial for functional genes like COI (Pentinsaari et al. 2016). Thus, positional VLF error rates are expected to be considerably different from error rates observed for entire sequences. Findings for birds like Canada goose may aid in explaining interesting population-level phylogeographic patterns consistent with colonisation of refugia during Pleistocene glaciations, such as founder events, bottlenecks, migrations and admixture within this and other groups (Scribner et al. 2003). Finally, a worthwhile and timely next step would be to assess errors in COVID-19 nucleotide sequence data as was done by Dunn (2021) using *VLF*.

## Conclusion

In this paper, we present a new R package, *VLF*, along with a simple R workflow, for quality assessment and curation of large reference sequence libraries through detection of sequence artifacts, such as machine errors, indels and NUMTs/pseudogenes, inconsistencies which have been observed in diverse COI sequence datasets for crayfish, grasshoppers, marine Metazoa and insects for instance (Song et al. 2008, Buhay 2009, Hebert et al. 2022, Schultz and Hebert 2022). Similar computational and statistical tools, in the form of MATLAB packages, R packages, Python packages and methodological pipelines, used to assess anomalies in DNA (meta)barcodes, have been released. Examples include divisive hierarchical clustering: *DADA* (Rosen et al. 2012) and *DADA2* (Callahan et al. 2016); artificial neural networks: (Ma et al. 2018); Profile Hidden Markov Models: *coil* (Nugent et al. 2020), *debar* (Nugent et al. 2021 and Porter and Hajibabaei 2021); distribution sample quantiles: *MACER* (Young et al. 2021); and Shannon entropy: *SequenceBouncer *(Dunn 2021), *A2G2* (Hleap et al. 2020), DnoisE (Antich et al. 2022 and Turon et al. 2020). These methods and programmes are beginning to see widespread use within the biodiversity and regulatory science communities. *VLF* brings several advantages over Stoeckle and Kerr (2012)'s method: our approach is simpler to implement, much faster to run and less prone to human error. Importantly, we stress the need to clean generated taxon sequence datasets as much as possible to mitigate the contribution of PCR/sequencing errors to specimen identification, particularly to the level of species and suggest steps to take in this regard. We have shown here various ways in which the *VLF *R package can be used to address interesting questions in evolutionary biology, molecular genetics, population genetics and phylogeography. As sequence cleaning forms a major part of the DNA barcoding effort, the availability of *VLF *as a first line of defence, should greatly facilitate integration of sequence error analysis and quality checking into a wide range of novel bioinformatics workflows. In fact, VLF analysis, in the form of alignment trimming at the 5’ and 3’ ends, has already begun to be incorporated into said pipelines, such as that of Loeza-Quintana and Adamowicz (2018) for the iterative calibration of Echinoderm molecular clocks, based on accurate timing of geologic events and that of May et al. (2020) to assess the effect of various ecological and environmental traits on molecular evolutionary rates in ray-finned fishes. Aside from purely biological applications of VLF analysis, we foresee widespread use of *VLF* in regulatory settings to ensure high accuracy of specimen identifications at large.

## Data availability

*VLF* version 1.1 is available for download through the Comprehensive R Archive Network (CRAN) directly within R using the successive commands:

>install.packages(“VLF”)

>library(VLF).

The reference manual for *VLF*, which includes built-in functions with explanations for their proper use, can be accessed by typing:

>?VLF.

The birds nucleotide and amino acid dataset used by Stoeckle and Kerr (2012) can be accessed by typing:

>data(birds)

>data(birds_aminoAcids)

Package source code can be accessed by typing the name of the desired function. Alternatively, code is accessible via GitHub at https://github.com/jphill01/VLF.R.

Raw (unaligned) 5’-COI sequences used in the forensic VLF analysis can be directly downloaded using the Project and Dataset Search field within the BOLD Workbench.

## Supplementary Material

EF0A9427-3ED5-5B59-B6B3-28B7813EC8BB 10.3897/BDJ.11.e96480.suppl1 Supplementary material 1 Fish 5'-COI FASTA Alignment Data type DNA sequences Brief description 652 bp FASTA alignment of 2371 published fish 5'-COI DNA barcodes. File: oo_743529.fas https://binary.pensoft.net/file/743529 Phillips, JD; Athey, TBT; McNicholas, PD; Hanner, RH

56543BF4-A013-5CA6-915A-3153E0733FA1 10.3897/BDJ.11.e96480.suppl2 Supplementary material 2 VLF analysis R script Data type R script Brief description R script to reproduce all analyses. File: oo_757483.R https://binary.pensoft.net/file/757483 Phillips, JD; Athey, TBT; McNicholas, PD; Hanner, RH

## Figures and Tables

**Table 1.** Specimen VLF distribution for birds.

**VLFs**	1	2	3	4	5	6	9	10	13	15
**Specimens**	446	63	27	3	2	5	2	2	1	1

**Table 2.** Positional VLFs for birds.

**VLFs**	1	2	3	4	5	6	7	8	9	10	11	13	14	15	18
**Positions**	91	53	25	19	12	11	5	7	5	3	4	2	2	1	1

**Table 3.** Positional error rates for birds. Per barcode error rates are indicated in parentheses.

**Singleton**	8.54 x 10^-5 ^(0.0553)
**Shared**	3.92 x 10^-5 ^(0.0254)
**Total**	1.25 x 10^-4 ^(0.0810)

**Table 4.** Specimen VLF distribution for Canada goose (*Branta Canadensis*).

**VLFs**	1	2	3
**Specimens**	11	5	2

**Table 5.** Positional VLF distribution for Canada goose (*Branta Canadensis*).

**VLFs**	1	2	3	4	10
**Positions**	5	1	2	1	1

**Table 6.** Positional error rates for Canada goose (*Branta canadensis*). Per barcode error rates are indicated in parentheses.

**Singleton**	7.74 x 10^-3 ^(5.016)
**Shared**	3.56 x 10^-3 ^(2.304)
**Total**	0.0113(7.320)

**Table 7.** Summary of public BOLD projects used in this study.

**BOLD Project Code**	**No. of 5’-COI Sequences**	**No. of Families/Genera/Species**
EBFSF	296	2/6/10
EMRKT	91	23/32/20
EWSHK	1050	18/32/76
SSNA	934	1/2/8
**Total**	**2371**	**44/72/114**

**Table 8.** Specimen VLF distribution for fishes.

**VLFs**	1	2	3	4	6	34
**Specimens**	42	10	1	3	1	1

**Table 9.** Positional VLF distribution for fishes.

**VLFs**	1	2	3
**Positions **	55	25	4

**Table 10.** Positional error rates for fishes. Per barcode error rates are indicated in parentheses.

**Singleton**	7.81 x 10^-5 ^(0.0509)
**Shared**	1.56 x 10^-5 ^(0.0102)
**Total**	9.37 x 10^-5 ^(0.0611)
